# Serum EZH2 is a novel biomarker for bladder cancer diagnosis and prognosis

**DOI:** 10.3389/fonc.2024.1303918

**Published:** 2024-02-27

**Authors:** Feng Li, Pengqiao Wang, Jun Ye, Guoping Xie, Jinfeng Yang, Wei Liu

**Affiliations:** ^1^ Department of Urology, The First Affiliated Hospital of Chengdu Medical College, Chengdu, China; ^2^ Department of Medical Administration, The First Affiliated Hospital of Chengdu Medical College, Chengdu, China; ^3^ Department of Clinical Laboratory, The Second Affiliated Hospital of Guizhou Medical University, Kaili, China; ^4^ Department of Gastrointestinal Surgery, The First Affiliated Hospital of Chengdu Medical College, Chengdu, China; ^5^ Department of Oncology, The First Affiliated Hospital of Chengdu Medical College, Chengdu, China

**Keywords:** EZH2, biomarker, bladder cancer, diagnosis, overall survival rate, progression-free survival rate

## Abstract

**Objective:**

The primary objective of this study was to examine the levels of serum EZH2 in patients diagnosed with bladder cancer, and subsequently evaluate its potential as a biomarker for both the diagnosis and prognosis of bladder cancer.

**Methods:**

Blood samples were obtained from 115 bladder cancer patients and 115 healthy persons. We measured the EZH2 concentrations in the serum of these subjects via enzyme-linked immunosorbent assay (ELISA). To assess the diagnostic performance of serum EZH2 in detecting bladder cancer, we plotted receiver operating characteristic (ROC) curves and calculated their corresponding area under the curve (AUC). We also used the Cox regression model and log-rank test to investigate the correlation between EZH2 levels and clinicopathological characteristics, and survival rates of bladder cancer patients.

**Results:**

Serum EZH2 levels were significantly higher in bladder cancer patients when compared to those in healthy persons. Serum EZH2 levels exhibited a significant correlation with TNM stage, lymph node metastasis, muscle invasion, and tumor size. At a cutoff value of 8.23 ng/mL, EZH2 was able to differentiate bladder cancer patients from healthy persons, with an AUC of 0.87, a sensitivity of 81.31%, and a specificity of 78.42%. High EZH2 levels correlated with poor overall survival rates and progression-free survival rates of bladder cancer patients.

**Conclusions:**

Serum EZH2 levels were elevated in bladder cancer patients, and patients with higher serum EZH2 levels exhibited a poorer prognosis. This indicates that serum EZH2 could be a novel biomarker for bladder cancer diagnosis and prognosis. Such findings could improve the prognosis of bladder cancer patients by facilitating early detection and continuous monitoring.

## Introduction

Bladder cancer is a frequently diagnosed malignancy, ranking fourth in global cancer diagnoses and eighth among causes of cancer-related deaths in men (1, 2). This disease is primarily divided into two categories: non-muscle invasive bladder cancer (NMIBC) and muscle-invasive bladder cancer (MIBC) (3, 4). Transurethral resection of the bladder tumor (TURBT) is the primary treatment for NMIBC, while MIBC is typically treated with a combination of radical cystectomy and cisplatin-based chemotherapy (3, 5). However, the long-term effectiveness of these treatments is significantly compromised due to tumor recurrence and resistance to chemotherapy (6, 7). Previous studies have shown that early diagnosis and continuous monitoring can improve the survival rates of bladder cancer patients (8, 9).

The early detection and continuous monitoring of bladder cancer have consistently presented challenges in the field of urology, largely due to the lack of highly sensitive and specific methods (10, 11). Although cystoscopy is widely utilized as the gold standard for identifying bladder cancer, it is not a viable screening method owing to its invasive nature and high cost (12). At present, urine exfoliative cytology serves as the standard screening technique for bladder cancer. It effectively detects high-grade bladder cancer but is less successful in identifying low-grade and early-stage cases (13). Furthermore, the accuracy of cytology is heavily dependent on the pathologist’s expertise, rendering it an inefficient screening test (13). While the US Food and Drug Administration has approved several urinary biomarkers, their usefulness has been limited due to low specificity and high data heterogeneity (14–16). Therefore, there is a pressing need to identify highly sensitive, specific, and noninvasive biomarkers for early diagnosis and continuous monitoring of bladder cancer.

EZH2 is a crucial component of the Polycomb Repressive Complex 2 (PRC2) and acts as a critical regulator in various cellular functions, including differentiation, proliferation, and stem cell renewal (17, 18). Its fundamental role in cellular biology highlights its importance in maintaining normal cellular processes. However, the dysregulation of EZH2 can lead to significant pathological consequences. Increasing evidence links the impaired EZH2 function to the onset and progression of various cancers, such as prostate, bladder, colorectal, and breast cancers (19–21). This connection is particularly noteworthy because it implies a critical role of EZH2 in cancer biology, influencing tumor initiation and progression. Previous studies indicate that EZH2 is overexpressed in bladder cancer tissues, playing an important role in tumor metastasis and recurrence. Additionally, studies have shown that inhibiting EZH2 activity can significantly suppress the proliferation and metastasis of bladder cancer cells (20, 22, 23). Therefore, EZH2 holds the potential as a diagnostic and prognostic biomarker for bladder cancer, offering new insights into the disease’s initiation, progression, and response to treatment.

In the present study, we examined the expression patterns of EZH2 in both bladder cancer tissues and serum samples of patients. We further assessed its diagnostic performance and prognostic implications for bladder cancer patients. The findings of our study may improve the prognosis of bladder cancer patients through early diagnosis and continuous monitoring.

## Patients and methods

### Study population

This research got its approval from the Ethics Committee of The First Affiliated Hospital of Chengdu Medical College (No. 21824535), and in strict compliance with the ethical guidelines of the Declaration of Helsinki. All participants provided their written informed consent. From October 2017 to May 2019, the study recruited patients who had newly been diagnosed with bladder cancer. Inclusion criteria were (1): over 18 years old (2); having a bladder cancer diagnosis confirmed by histopathological examinations (3); not having received any anti-cancer treatments such as chemotherapy or radiotherapy (4); having no other malignancies, and (4) possessing complete patient information. Patients with conditions like diabetes, autoimmune diseases, organ failures, or hematological diseases were excluded from the study. Ultimately, a total of 115 bladder cancer patients were included in the study.

Diagnoses of bladder cancer were made based on histological findings, and tumor stages were determined using the World Health Organization’s (WHO) tumor-node-metastasis (TNM) staging system (24). The study hospital has a physical examination center that offers physical examination services to individuals. These services include the collection of medical history, ultrasound examinations for the urinary system and liver, tumor marker tests, liver and renal function tests, CT scans, urine analysis, urine exfoliative cytology, and so on. We conducted a review of the physical examination reports and selected 115 healthy individuals as control subjects for the study. These control individuals displayed normal results on physical examination reports and had no history of malignancy, bladder disease, or other urological conditions. Demographics between bladder cancer patients and healthy control subjects were present in **Supplementary Table 1**.

The definition of disease progression adheres to the guidelines established by the International Bladder Cancer Group. For non-muscle invasive bladder cancer, progression is defined as an increase in T stage from CIS or Ta to T1; development of ≥T2 or lymph node (N+) disease or distant metastasis (M1); or an increase in grade from low to high. In the case of muscle-invasive bladder cancer, progression after treatment is defined by a tumor relapse with pathological evidence in the bladder with tumor stage ≥pT2 or distant relapse as N1-3 or M1 (25, 26).

### Data collection and follow−up

Clinicopathologic data and demographic data were retrieved from the electronic medical record system of the study hospital. After a 12-hour fasting period, blood samples were collected from all participants. These samples were then centrifuged at 2000 g for 5 minutes at room temperature to isolate the serum. The resulting serum samples were transferred into Eppendorf tubes and preserved at -80°C refrigerator for future analysis. After collecting all the serum samples, they were analyzed in the same batch. EZH2 concentrations in serum samples were determined with a commercially available ELISA kit according to the protocol of the manufacturer.

The overall survival (OS) refers to the survival duration from the initial diagnosis to either the date of death from any cause or the date of the last follow-up. The progression-free survival (PFS) was defined as the period during which patients with bladder cancer live without disease progression or death after treatment. The follow-up protocol was structured with bi-monthly visits for the first two years, followed by quarterly visits for the next two years, and then semi-annual visits thereafter. The follow-up period ended in July 2023.

### Immunohistochemistry

Tissue specimens were immersed in a 4% paraformaldehyde solution and kept at room temperature for a duration of two days. Then they were embedded in paraffin and sliced into sections with a 4 μm thickness. These sections underwent dewaxing and dehydration, followed by antigen retrieval immersing in a citric acid solution. The sections were blocked by 10% goat serum to inactivate endogenous peroxidases and eliminate the impact of non-specific staining.

They were then incubated with primary antibody of anti-EZH2 which was obtained from Abcam. We didn’t stain the primary antibody when we set negative control. After washed three times with PBS, the sections were incubated with an HRP-labeled goat anti-mouse secondary antibody. After another three times PBS washing, the sections were stained with diaminobenzidine to display the expression intensity. The EZH2 expression intensity were analyzed by pathologists under a Zeiss LSM500 microscope (Zeiss International, Oberkochen, Germany).

### Statistical analysis

Statistical analyses were carried out by using the SPSS software (Version 31.0, Chicago, IL, USA). For continuous data, the independent-sample t-test was applied, while the chi-square test or Fisher’s exact test was used for categorical data. The receiver operating characteristic (ROC) curve was employed to assess the diagnostic performance of serum EZH2 levels for identifying bladder cancer. We used the Kaplan–Meier method to plot the survival curves, and the log-rank test was used to analyze the statistical significance between the two curves. Finally, to identify prognostic factors associated with bladder cancer survival, we performed both univariate and multivariate Cox proportional hazards regression analyses. We checked the fitness of the Cox regression model with Cumulative Sums of Martingale-Based Residuals as described in the previous study (27).

## Results

### EZH2 expression levels were elevated in both bladder cancer tumor tissues and serum samples of bladder cancer patients

**Figure 1:**
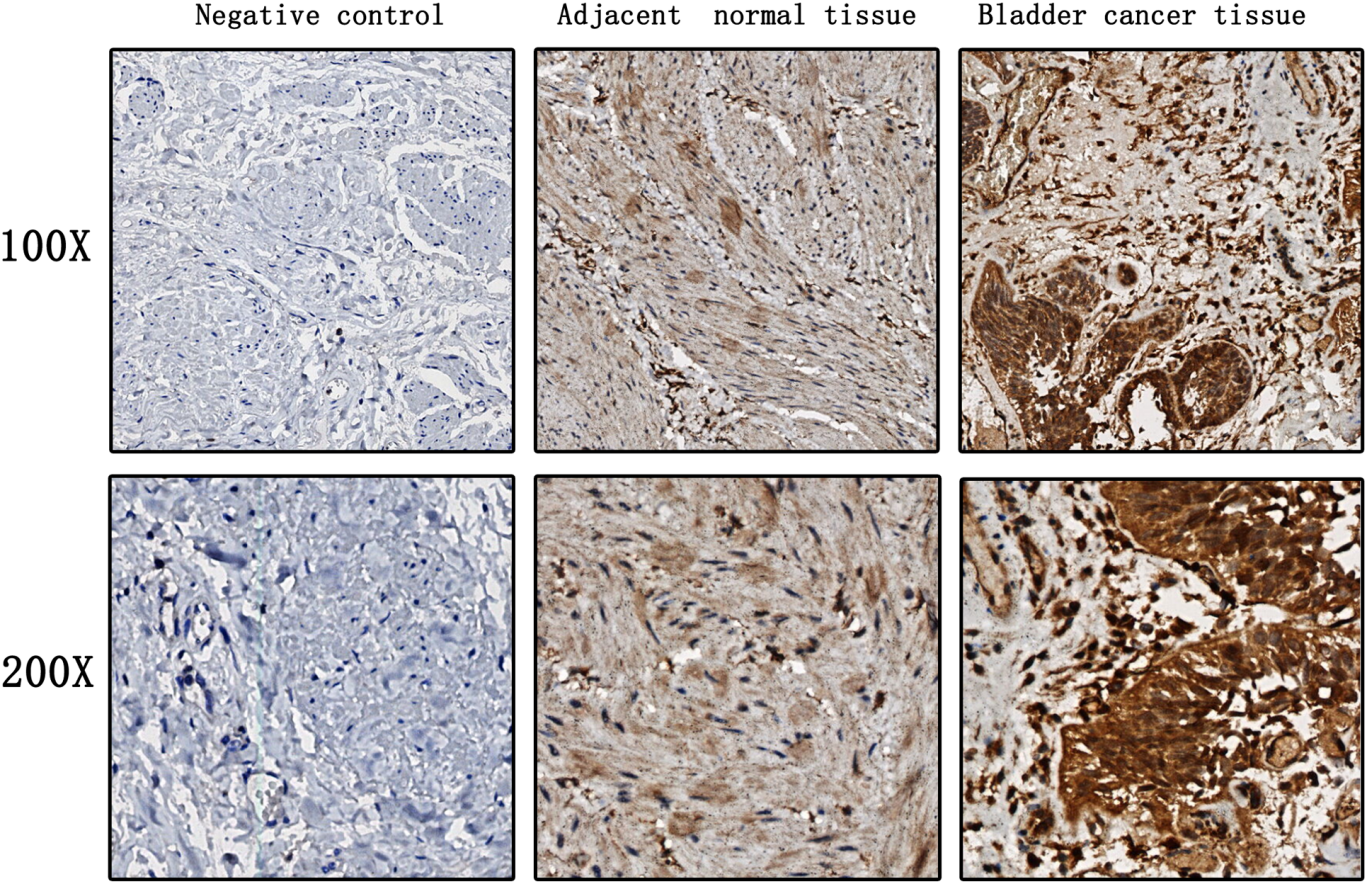
EZH2 expression levels in bladder cancer tumor tissues and adjacent normal bladder tissues.

**Figure 2:**
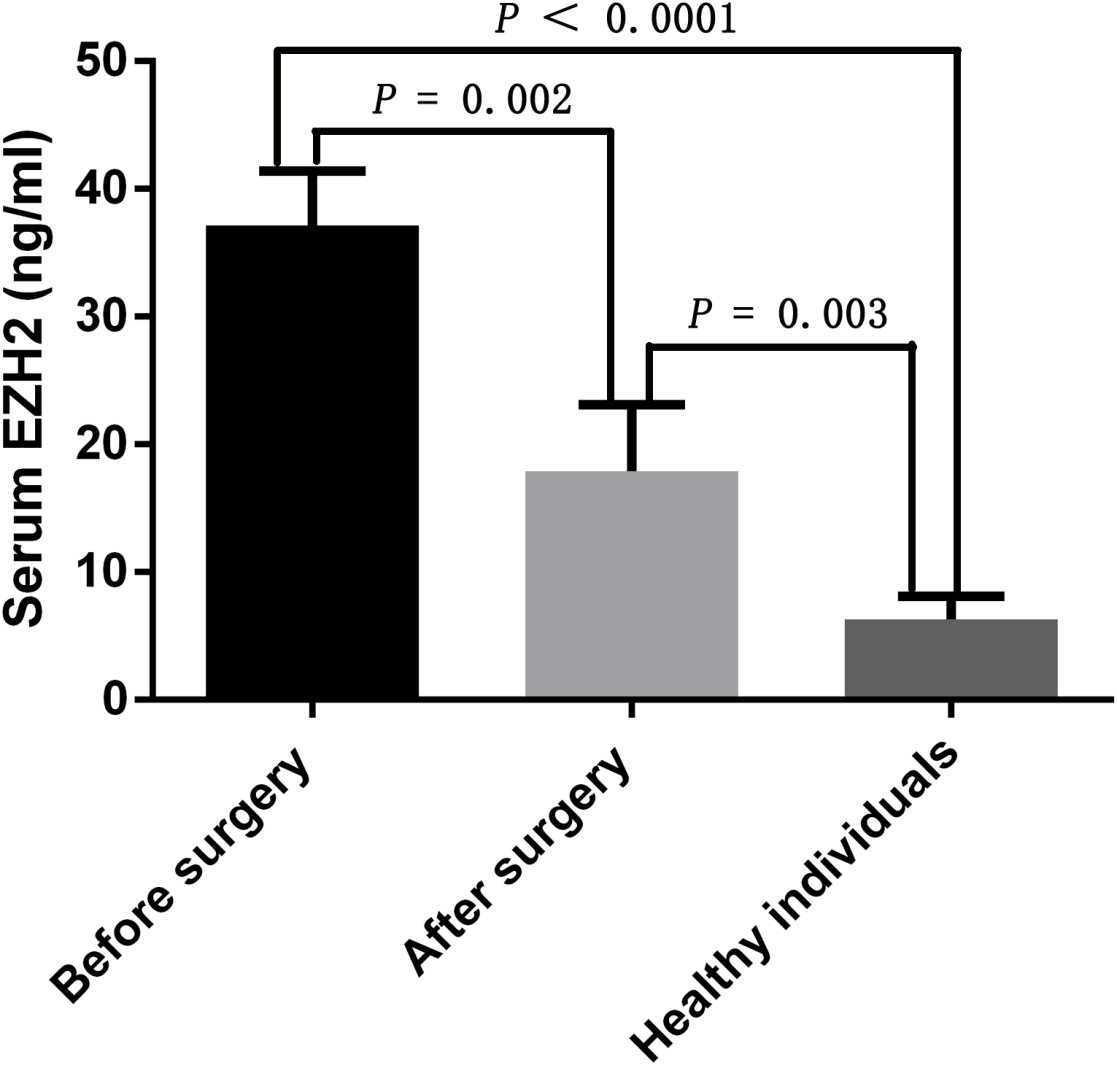
EZH2 expression levels in the serum of bladder cancer patients before and after surgery, and healthy control individuals. An independent-sample t test was used to analyze the statistical significance between the two groups. A *p* < 0.05 was considered statistically significant.

EZH2 expression levels were significantly higher in bladder cancer tumor tissues when compared to those in adjacent normal bladder tissues (**Figure 1**). Moreover, EZH2 levels in the serum of bladder cancer patients were significantly higher than those in healthy control individuals, and the median serum EZH2 levels were 36.23 ng/ml and 8.12 ng/ml, respectively (**Figure 2**). In order to confirm that serum EZH2 originated from the bladder cancer tumor tissues, we compared serum EZH2 levels before and after the surgical resection of bladder cancer tumors. Blood samples were collected from bladder cancer patients one week following their surgeries. It showed that EZH2 levels were significantly decreased after surgery, although they remained significantly higher than levels observed in healthy control individuals (**Figure 2**).

### Correlations between EZH2 levels and clinicopathological characteristics of bladder cancer patients

To determine the association between serum EZH2 levels and clinicopathological characteristics of categorical variables, bladder cancer patients were divided into two groups based on the median EZH2 levels (**Table 1**). Serum EZH2 levels were considered as continuous variables when compared with clinicopathological characteristics of continuous variables (**Table 2**). The findings revealed that EZH2 levels were significantly correlated with clinicopathological characteristics including TNM stage, lymph node metastasis, muscle invasion, and tumor size (**Tables 1**, **2**). However, there was no significant correlation between EZH2 levels and other clinicopathological characteristics such as gender, tumor differentiation, age, height, weight, and BMI (**Tables 1** , **2**).

**Table 1:** Correlations between EZH2 levels and clinicopathological characteristics of bladder cancer patients for categorical variables.

Characteristics	Total (115)	EZH2 levels		*p-value*
		Low (62)	High (53)	
**Gender**				0.718
Male	65	36	29	
Female	50	26	24	
**Tumor differentiation**				0.670
Well and moderate	67	35	32	
Poor	48	27	21	
**TNM stage**				**0.001**
I+ II	58	40	18	
III+ IV	57	22	35	
**Lymph node metastasis**				**< 0.0001**
No	64	45	19	
Yes	51	17	34	
**Muscle invasion**				**0.003**
No	54	37	17	
Yes	61	25	36	

A chi-square test analyzed the statistical significance. A p <0.05 was considered statistically significant and marked in bold text.

**Table 2:** Correlations between EZH2 levels and clinicopathological characteristics of bladder cancer patients for continuous variables.

Variables	Serum EZH2 levels (ng/ml)	
	*r*	*p-value*
Age (year)	-0.13	0.637
Height (cm)	-0.27	0.361
Weight (kg)	0.25	0.194
BMI (kg/m^2^)	0.34	0.219
Tumor size (cm)	0.68	< **0.0001**

Data are analyzed by Pearson correlation analysis. A p-value < 0.05 was considered statistically significant and marked in bold text.

### Diagnostic performance of serum EZH2 levels for detecting bladder cancer

**Figure 3:**
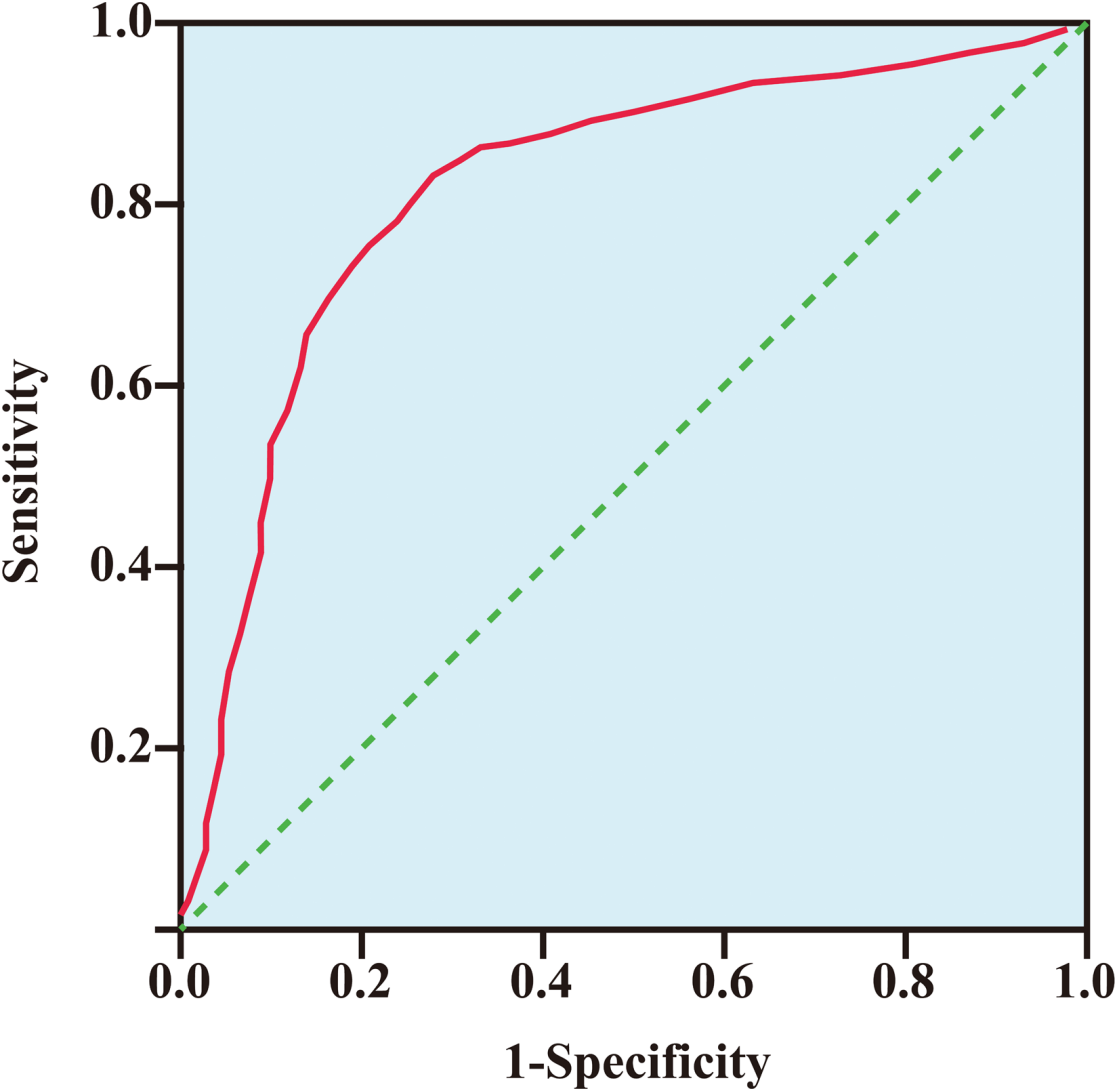
The receiver operating characteristic curve of EZH2 level for detecting bladder cancer. The area under the curve (AUC) was 0.87 (95% CI: 0.526–0.963, p < 0.001), with a sensitivity of 81.31% and a specificity of 78.42%.

A total of 115 bladder cancer patients and 115 healthy control individuals were included in ROC curve analyses to assess the diagnostic performance of serum EZH2 levels for detecting bladder cancer. The data showed that at an optimal cut-off value of 8.23 ng/ml, EZH2 levels could effectively distinguish bladder cancer patients from healthy individuals. The area under the ROC curve (AUC) was 0.87 (95% CI: 0.526–0.963, p < 0.0001), with a sensitivity of 81.31% and a specificity of 78.42% (**Figure 3**).

### Identification of risk factors for overall survival rate and progression-free survival rate of bladder cancer patients

We used both univariate and multivariate Cox proportional hazards regression analyses to identify prognostic factors for bladder cancer patients. The univariate analysis showed that factors like tumor differentiation, lymph node metastasis, muscle invasion, age, tumor size, and EZH2 levels were risk factors for overall survival rates (**Table 3**). When adjusting for potential confounding factors, including age, tumor differentiation, tumor size, lymph node metastasis, and muscle invasion, the multivariate analysis identified the muscle invasion (HR = 3.25, 95%Cl: 1.48–5.42, *p* = 0.021), tumor size (HR = 2.24, 95%Cl: 1.52–3.42, *p* = 0.004), and EZH2 levels (HR = 3.07, 95%Cl: 2.11–5.09, *p* = 0.006) as independent risk factors for overall survival rates (**Table 3**).

**Table 3:** Identification of risk factors for overall survival rate using a Cox regression model.

Characteristics	Univariate analysis	*p-value*	Multivariate analysis	Adjusted *p-value*
	HR (95%CI)				HR (95%CI)
**Gender**				
Male	1.00 (Reference)		1.00 (Reference)	
Female	0.87 (0.49–2.26)	0.191	1.04 (0.72–2.67)	0.215
**Tumor differentiation**				
Well and moderate	1.00 (Reference)		1.00 (Reference)	
Poor	1.24 (0.53-2.61)	**0.014**	0.91 (0.42-2.71)	0.151
**Lymph node metastasis**				
No	1.00 (Reference)		1.00 (Reference)	
Yes	1.57 (1.13-2.16)	**0.014**	1.16 (0.62-2.21)	0.102
**Muscle invasion**				
No	1.00 (Reference)		1.00 (Reference)	
Yes	4.35 (2.47-6.31)	**0.017**	3.25 (1.48-5.42)	**0.021**
**Age** (year)	2.49 (1.02–4.79)	**0.001**	1.07 (0.57–2.98)	0.086
**Height** (cm)	1.18 (0.82–2.93)	0.247	0.86 (0.43–2.74)	0.172
**Weight** (kg)	1.19 (0.42-2.48)	0.421	0.95 (0.62-2.37)	0.427
**BMI** (kg/m^2^)	0.85 (0.47-2.13)	0.092	1.18 (0.81-2.92)	0.532
**Tumor size** (cm)	3.73 (2.05-6.03)	**0.0001**	2.24 (1.52-3.42)	**0.004**
**EZH2 levels** (ng/ml)	3.51 (1.29-5.28)	**0.0002**	3.07 (2.11-5.09)	**0.006**

CI, Confidence interval; HR, hazard ratio. Adjusted for potential confounding factors, including age, tumor differentiation, tumor size, lymph node metastasis, and muscle invasion. A p < 0.05 was considered statistical significance and marked in bold text.

We further evaluated progression-free survival rates, and found that factors like tumor differentiation, lymph node metastasis, muscle invasion, tumor size, and EZH2 levels were risk factors for progression-free survival rates (**Table 4**). Upon adjusting for confounding factors, including, tumor differentiation, tumor size, lymph node metastasis, and muscle invasion, only the tumor differentiation (OR = 1.84, 95%Cl: 1.08–3.92, *p* = 0.013), muscle invasion (OR = 2.51, 95%Cl: 1.18–4.62, *p* = 0.001), and EZH2 levels (OR = 2.48, 95%Cl: 1.33–4.24, *p* = 0.008) emerged as independent risk factors for progression-free survival rates (**Table 4**).

**Table 4:** Identification of risk factors for progression-free survival rate using a Cox regression model.

Characteristics	Univariate analysis	*p-value*	Multivariate analysis	Adjusted *p-value*
	HR (95%CI)		HR (95%CI)	
**Gender**				
Male	1.00 (Reference)		1.00 (Reference)	
Female	1.05 (0.82–2.04)	0.083	0.91 (0.63–2.31)	0.126
**Tumor differentiation**				
Well and moderate	1.00 (Reference)		1.00 (Reference)	
Poor	2.37 (0.68-3.74)	**0.001**	1.84 (1.08-3.92)	**0.013**
**Lymph node metastasis**				
No	1.00 (Reference)		1.00 (Reference)	
Yes	1.84 (1.13-3.72)	**0.007**	0.92 (0.63-1.57)	0.256
**Muscle invasion**				
No	1.00 (Reference)		1.00 (Reference)	
Yes	3.72 (1.35-5.42)	**0.005**	2.51 (1.18-4.62)	**0.001**
**Age** (year)	0.92 (0.61–1.53)	0.304	1.15 (0.72–1.45)	0.154
**Height** (cm)	0.84 (0.41–1.51)	0.076	1.18 (0.83–1.61)	0.254
**Weight** (kg)	1.14 (0.81-2.56)	0.576	0.81 (0.44-1.73)	0.092
**BMI** (kg/m^2^)	0.92 (0.53-1.57)	0.264	1.05 (0.74-1.77)	0.346
**Tumor size** (cm)	2.82 (1.81-5.24)	**0.002**	1.15 (0.82-1.61)	0.094
**EZH2 levels** (ng/ml)	4.32 (2.18-6.13)	**0.003**	2.48 (1.33-4.24)	**0.008**

CI, Confidence interval; HR, hazard ratio. Adjusted for confounding factors, including, tumor differentiation, tumor size, lymph node metastasis, and muscle invasion. A p < 0.05 was considered statistical significance and marked in bold text.

### Survival differences between bladder cancer patients with high serum EZH2 levels and low serum EZH2 levels

**Figure 4:**
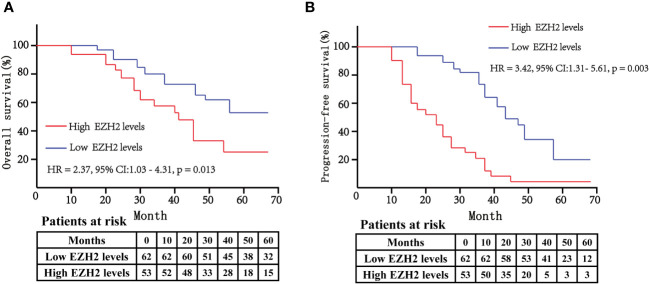
The Kaplan–Meier curves were used to compare the overall survival rate **(A)** and progression-free survival rate **(B)** between high-EZH2 group and low-EZH2 group. The log-rank test was used to analyze the statistical significance between the two curves. A p < 0.05 was considered statistically significant.

The above findings indicated that EZH2 was an independent risk factor for both overall survival rate and progression-free survival rate of bladder cancer patients. We further used Kaplan–Meier analysis to assess the survival difference between bladder cancer patients with high serum EZH2 levels and low serum EZH2 levels based on the median EZH2 levels. The data showed that the high-EZH2 group had significantly lower overall survival rate compared to the low-EZH2 group (HR: 2.37, 95% CI: 1.03–4.31, *p* = 0.013) (**Figure 4A**). Additionally, we assessed progression-free survival rate and found that the high-EZH2 group had lower progression-free survival rate than the low-EZH2 group (HR: 3.42, 95% CI: 1.31–5.61, *p* = 0.003) (**Figure 4B**).

## Discussion

Bladder cancer is one of the most aggressive and deadly malignancies (1, 2). The poor survival rates of bladder cancer patients may be due to the lack of early detection and continuous monitoring (28, 29). Imaging examinations and cystoscopy are commonly used methods for diagnosing bladder cancer and detecting its recurrence. They are standard clinical procedures, but they are not viable for large-scale screening due to their invasive nature and high cost (12). Numerous studies and cancer prevention guidelines recommend that early diagnosis and continuous monitoring are essential for effectively reducing bladder cancer related mortality (30–32). The serum levels of tumor markers are closely correlated with the onset, progression, and recurrence of tumors (30). Therefore, identifying new biomarkers with high sensitivity and specificity for both early detection and continuous monitoring of bladder cancer holds the potential to significantly improve patient survival rates.

In this study, we found that EZH2 expression levels were significantly elevated in bladder cancer tumor tissues and serum samples of bladder cancer patients (**Figure 2**). Moreover, at an optimal cut-off value of 8.23 ng/ml, EZH2 could effectively distinguish bladder cancer patients from healthy individuals, with an AUC of 0.87 (95% CI: 0.526–0.963, *p* < 0.0001), a sensitivity of 81.31%, and a specificity of 78.42% (**Figure 3**). These findings suggested that serum EZH2 levels had a good diagnostic performance for detecting bladder cancer. Given the efficiency, convenience, and cost-effectiveness of serum tumor markers in cancer screening, serum EZH2 holds the potential as a biomarker for bladder cancer screening (33). It could be helpful in facilitating the early detection of bladder cancer.

We also found that following the surgical removal of bladder tumors, the serum levels of EZH2 showed a significant decline (**Figure 2**). This indicates that the elevated serum EZH2 levels might indeed stem from the bladder tumor tissues. However, even after surgery, serum EZH2 levels in bladder cancer patients remained notably higher when compared to those in healthy individuals (**Figure 2**). This discrepancy could be attributed to the short one-week period between the surgery and the subsequent postoperative blood sample collection. Such a brief interval might not provide adequate time for the complete degradation of the circulating EZH2 protein in the blood. On the other hand, the sustained high levels of serum EZH2 after surgery could be attributed to residual tumor cells still circulating in the blood stream, which have not been fully eradicated by the immune system or chemotherapy treatments. It’s important for future research to investigate the typical duration required for EZH2 levels to normalize after surgery, as this could help monitor tumor recurrence.

The recurrence rate of bladder cancer after surgery is high, especially for muscle-invasive bladder cancer (34). It is currently recognized that tumor differentiation, tumor size, lymph node metastasis, and pathological stage are risk factors for predicting bladder cancer recurrence (35, 36). In this study, we found that serum EZH2 levels were significantly associated with factors including TNM stage, tumor size, muscle invasion, and lymph node metastasis (**Tables 1**, **2**). This indicates that serum EZH2 could be a meaningful biomarker for predicting the recurrence of bladder cancer. This study also demonstrated that bladder cancer patients with higher serum EZH2 levels had lower overall survival rate and progression-free survival rate than patients with lower serum EZH2 levels (**Figure 4**). These findings suggest that serum EZH2 could serve as a biomarker for predicting prognosis in patients with bladder cancer. Therefore, it suggests that patients with high serum EZH2 levels should receive close post-surgical monitoring to detect any signs of recurrence or progression. Overlooking such indicators might result in some patients developing muscle-invasive bladder cancer, ultimately necessitating a radical cystectomy and having a poor prognosis (37, 38).

Several biomarkers have been approved by the FDA for the diagnosis and follow-up of bladder cancer, such as BTA TRAK, NMP22, NMP22, and BTA *stat (* 30). However, they have not yet been integrated into clinical guidelines. The main reason for this is their absence in current clinical decision-making processes. As a result, the potential additional benefits of these molecular markers in diagnosing bladder cancer are still unverified. Therefore, there is an urgent need to develop protocols and conduct prospective trials to establish a foundation for the future integration of these molecular markers into clinical decision-making. Our preliminary study found that serum EZH2 has the potential to be a biomarker for bladder cancer diagnosis and prognosis. However, further studies are needed to validate the findings.

Compared to cystoscopy and imaging methods, assessing serum EZH2 offers benefits such as simplicity, speed, reproducibility, non-invasiveness, and cost-effectiveness. It could be valuable for large-scale patient screening and risk stratification before surgery, guiding both the selection of treatment plans and post-operative monitoring. However, our research has its limitations that should be acknowledged. Firstly, as the study was carried out in a single medical center, the results may not be generalized to other medical centers. Secondly, the study population and control population were relatively small, and the selection bias couldn’t be completely ruled out. Thirdly, further large-scale population-based prospective studies are needed to confirm the efficiency of serum EZH2 as a biomarker for bladder cancer screening and monitoring. Fourthly, we did not explore the underlying mechanisms that associated with the elevated levels of serum EZH2 in bladder cancer patients, which is a field that deserves further study.

## Conclusions

Serum EZH2 levels were elevated in bladder cancer patients, and patients with higher serum EZH2 levels exhibited a poorer prognosis. This indicates that serum EZH2 could be a novel biomarker for bladder cancer diagnosis and prognosis. Such findings could improve the prognosis of bladder cancer patients by facilitating early detection and continuous monitoring. Nonetheless, further research is needed to fully understand its clinical significance and potential contribution to bladder cancer care.

## Data availability statement

The original contributions presented in the study are included in the article/**Supplementary Material**. Further inquiries can be directed to the corresponding authors.

## Ethics statement

The studies involving humans were approved by Ethics Committee of The First Affiliated Hospital of Chengdu Medical College (No. 21824535). The studies were conducted in accordance with the local legislation and institutional requirements. The human samples used in this study were acquired from a by- product of routine care or industry. Written informed consent for participation was not required from the participants or the participants’ legal guardians/next of kin in accordance with the national legislation and institutional requirements.

## Author contributions

FL: Writing – original draft, Investigation. PW: Data curation, Validation, Writing – review & editing. JY: Writing – review & editing, Formal analysis, Methodology. GX: Writing – review & editing, Funding acquisition, Supervision. JFY: Validation, Investigation, Writing – review & editing. WL: Supervision, Conceptualization, Writing – original draft.
