# Supplementary material for: Serum EZH2 is a novel biomarker for bladder cancer diagnosis and prognosis
Source: Front Oncol. 2024 Feb 27;14:1303918. doi: 10.3389/fonc.2024.1303918 (PMC10927824; doi:10.3389/fonc.2024.1303918)
Supplement: Supplementary file 1 [file Table_1.docx]

**Supplement Table 1.** Demographics between bladder cancer patients and healthy control subjects.

| Variables | bladder cancer patients | healthy control subjects | *p-value* |
| --- | --- | --- | --- |
|  | (n=115) | (n=115) |  |
| Age (years) | 65±3.2 | 64±2.7 | 0.091 |
| Height (cm) | 170±5.3 | 171±5.6 | 0.217 |
| Weight (kg) | 66±6.2 | 68±6.7 | 0.132 |
| BMI (kg/m^2^) | 24±2.6 | 23±3.1 | 0.341 |
| **Gender** |  |  | 1.00 |
| Male | 65 | 65 |  |
| Female | 50 | 50 |  |

**Notes:** age, height, weight, and BMI were analyzed by the independent sample t-test, while gender was analyzed by chi-square test.
